# Asociación entre sobrepeso/obesidad infantil y pobreza en la Región Pacífica y Bogotá, Colombia; 2018: un estudio ecológico mixto

**DOI:** 10.15446/rsap.V25n3.105820

**Published:** 2023-05-01

**Authors:** Juana C. Calero-Malo, Sandra M. Montoya-Sanabria

**Affiliations:** 1 JC: MD. M. Sc. Salud Pública. Instituto de Salud Pública, Pontificia Universidad Javeriana, Bogotá, Colombia calerojc@javeriana.edu.co Pontificia Universidad Javeriana Instituto de Salud Pública Pontificia Universidad Javeriana Bogotá Colombia calerojc@javeriana.edu.co; 2 SM: RN. M.Sc. Ph. D. Salud Pública. Profesora Asistente, Instituto de Salud Pública. Pontificia Universidad Javeriana. Bogotá, Colombia montoya.smilena@javeriana.edu.co Pontificia Universidad Javeriana Instituto de Salud Pública Pontificia Universidad Javeriana Bogotá Colombia montoya.smilena@javeriana.edu.co

**Keywords:** Obesidad, obesidad pediátrica, sobrepeso infantil, pobreza *(fuente: DeCS, BIREME)*, Obesity, pediatric obesity, overweight, poverty *(source: DeCS, NLM)*

## Abstract

**Objetivo:**

Estimar la asociación entre pobreza y sobrepeso/obesidad en población de 5 a 19 años en la Región Pacífica y en Bogotá D. C. (Colombia).

**Material y Métodos:**

Estudio ecológico mixto con análisis retrospectivo de series de tiempo (2015-2019) de la prevalencia de sobrepeso/ obesidad en la Región Pacífica y en Bogotá D. C. Se hizo un análisis de regresión múltiple, empleando modelos de regresión binomial negativa de datos panel de efectos fijos, para analizar la asociación el sobrepeso/obesidad y las necesidades básicas insatisfechas en las entidades territoriales de interés.

**Resultados:**

Se estimaron prevalencias de obesidad de 133 (Cauca) a 1 127 (Bogotá D. C.) casos por 100 000 habitantes. Los hogares con condiciones de miseria y pobreza en los departamentos de Nariño (OR 1,6 IC 1,6-1,7) y Chocó (OR 1,5 IC 1,5-1,6) tuvieron mayor riesgo de sobrepeso/obesidad que los demás territorios analizados.

**Discusión:**

El sobrepeso/obesidad es un problema social dada su naturaleza multifactorial. Para mejorar la comprensión debe considerarse la influencia de dimensiones como la clase social y la etnia.

## Abstract (en)

**Objective:**

To estimate the association between poverty and overweight/obesity in the population between 5 and 19 years of age in the Pacific Region and Bogotá D.C. -Colombia.

**Material and Methods:**

Mixed ecological study with retrospective time series analysis (2015-2019) of the prevalence of overweight/obesity in the Pacific Region and Bogotá D.C. and analysis of the Unsatisfied Basic Needs Index in 2018. We developed a multiple regression analysis, using fixed effects panel data negative binomial regression models to analyze the association between overweight/obesity and unsatisfied basic needs in the interested territorial entities.

**Results:**

Obesity prevalences were estimated from 133 (Cauca) to 1,127 (Bogotá D.C.) cases per 100,000 inhabitants. Households with conditions of misery and poverty in the departments of Nariño (OR 1.6 IC 1.6 -1.7) and Chocó (OR 1.5 IC 1.5-1.6) had a higher risk of overweight/obesity than the other territories analyzed.

**Discussion:**

Overweight/obesity is a social problem given its multifactorial nature. To improve understanding, the influence of dimensions such as social class and ethnicity should be considered.

La obesidad infantil es uno de los principales problemas de salud pública del siglo XXI. Entre el 2000 y el 2013 los casos aumentaron de 32 a 42 millones a escala mundial 1. Esta condición tiene implicaciones importantes en la vida adulta, dado que se asocia con enfermedades crónicas no transmisibles (ECNT) como la hipertensión arterial y la diabetes mellitus, y con otras condiciones como la apnea del sueño o las alteraciones ortopédicas, además de afectar también las habilidades sociales y el desempeño escolar 2. Estas dificultades disminuyen la calidad de vida y aumentan la mortalidad y los gastos de los sistemas de salud.

Esta enfermedad, entendida como constructo socio-cultural y no solo como producto de una alteración biológica, representa un panorama alarmante, ya que un niño obeso tiene entre un 77 % y 92 % más riesgo de ser también obeso en la adultez 3. En México, se calculó que "al disminuir un 1 % la prevalencia del índice de masa corporal, se podrían ahorrar 43 millones de dólares en atención médica en el 2030 y de 85 millones de dólares al 2050" 3,4. En Colombia, según datos de la Encuesta Nacional de Situación Nutricional (ENSIN) 2015, el promedio nacional de obesidad correspondía al 18,7 %, siendo mayor en la Región Pacífica (21 %) e inferior en la ciudad de Bogotá (16,7 %) 5.

El sobrepeso/obesidad se considera de origen multi-causal; entremezcla factores individuales y sociales, entre los cuales la dieta es el más importante, aun más que el ejercicio físico 6. No obstante, la transformación demográfica y epidemiológica de los últimos años ha influido en el aumento del consumo de alimentos ultra-procesados 7. Más allá de elecciones individuales condicionadas por la cultura y los medios de comunicación, la alimentación inadecuada es ocasionada, en parte, por dificultades en el acceso a alimentos saludables que permitirían tener una dieta balanceada y nutritiva 6.

Generalmente, los hogares gastan entre un 60 % y un 80 % de sus ingresos en alimentación 8. Si estos recursos se ven afectados o los precios aumentan y las personas no pueden costearse alimentos de calidad, los hogares se ven obligadas a adquirir alimentos de baja calidad, tales como comidas industrializadas, con alto contenido en sodio, azúcares, colorantes, conservantes, ricas en grasas saturadas, lo que incide en el desarrollo de sobrepeso/obesidad 9. El Instituto Nacional de Salud Pública de México, utilizando datos de la Encuesta Nacional de Salud en México (Ensanut), demostró que los niveles de sobrepeso/obesidad disminuían a medida que las condiciones socioeconómicas mejoraban 10. Similares hallazgos fueron reportados en un estudio realizado en el 2012 en Medellín, con una muestra de 5 556 adultos entre los 18 y los 64 años, donde se encontró una relación positiva entre sobrepeso/obesidad y un bajo nivel socioeconómico 11.

A pesar de las diferentes estrategias promulgadas por la Organización Mundial de la Salud (OMS) e implementadas por los gobiernos regionales (promoción de hábitos de vida saludable, regulación de precios de alimentos, control de la publicidad, impuestos a bebidas azucaradas, creación de espacios para realizar actividad física), es evidente la prevalencia de las ECNT y del sobrepeso/obesidad. Por ello, el estudio de las asociaciones y las posibles causas que puedan influir en tener una dieta inadecuada, se considera relevante en el campo de la salud pública, ya que la obesidad es el resultado de un complejo entramado que condiciona los comportamientos individuales 12. Por lo anterior, el objetivo de este estudio consistió en explorar la asociación entre la pobreza y la obesidad infantil, en niños, niñas y adolescentes (NNA) entre 5 y 19 años en la Región Pacífica y en Bogotá D. C. (Colombia).

## MATERIAL Y MÉTODOS

### Diseño y fuentes

**Figura 1:**
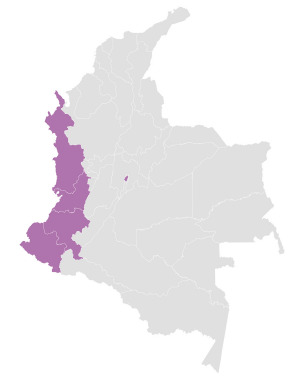
Mapa de Colombia, Región Pacífica y Bogotá. El mapa representa los departamentos de la Región Pacífica y la ciudad de Bogotá.

Este fue un estudio descriptivo, ecológico mixto con análisis retrospectivo de series de tiempo (2015-2019) de la prevalencia de sobrepeso/ obesidad en NNA residentes en la Región Pacífica (constituida por los departamentos de Chocó, Nariño, Valle del Cauca, Cauca) y en Bogotá Distrito Capital (DC) (Figura 1).

Se utilizó información de los registros integrales de prestación de servicios (RIPS), o registros de atención en salud, de la bodega de datos del Sistema de Información para la Protección Social (Sispro) 13 y los microdatos del Censo Nacional de Población y Vivienda (CNPV) realizado en Colombia en el 2018 14.

### Variables

Para la caracterización de la prevalencia de la obesidad en la población entre 5 y 19 años, las variables objeto de estudio fueron: edad, lugar de residencia, número de atenciones por año, número de personas atendidas por año, costo de la atención por año, tipo de atención (urgencias o consulta externa), año de atención y diagnóstico obesidad/sobrepeso. Solo se consideraron los servicios de atención ambulatoria, debido a que los diagnósticos de egreso hospitalario correspondían a "enfermedades endocrinas, nutricionales y metabólicas".

Para la medición directa de la pobreza se estimó el índice de necesidades básicas insatisfechas (NBI), a partir de cinco indicadores simples: vivienda inadecuada, viviendas con servicios públicos inadecuados, hogares en hacinamiento crítico, hogares con inasistencia escolar en niños en edad escolar y hogares con alta dependencia económica 15.

### Recolección de la información, procesamiento y análisis

En la identificación de atenciones de NNA diagnosticados con sobrepeso/obesidad se consideraron los códigos CIE10: E660 (obesidad debido a exceso de calorías), E668 (otros tipos de obesidad), E662 (obesidad extrema con hipoventilación alveolar), E669 (obesidad no especificada), E678 (otros tipos de hiperalimentación especificada), E65X (adiposidad localizada) y E68X (secuelas de hiperalimentación).

Se estimó la prevalencia de sobrepeso/obesidad correspondiente al 2018 utilizando los datos obtenidos del número de personas atendidas anualmente y la proyección de número de habitantes llevada a cabo por el Departamento Administrativo Nacional de Estadística (DANE), excluyendo los municipios que no reportaron NNA atendidos con estos diagnósticos. La construcción del NBI se realizó a partir de los cinco indicadores simples 15 señalados en la Tabla 1.

**Tabla 1:** Dimensión, variables censales del índice de necesidades básicas insatisfechas y su definición

Dimensión	Variables censales	Tipo de variable	Definición
Viviendas inadecuadas	Material predominante en paredes	Categórica	Viviendas móviles, o refugios naturales o bajo puentes o sin paredes. Cabecera municipal: tuviera pisos de tierra. Zona rural: piso de tierra y paredes en materiales de desecho o perecederos
	Material predominante en pisos	Categórica	
	Tipo de vivienda	Categórica	
Hacinamiento crítico	Número de cuartos	Continua	Más de tres personas por cuarto, excluyendo el garaje, la cocina y el baño
	Total personas en el hogar	Continua	
Servicios inadecuados	Acueducto	Categórica	Cabeceras municipales: hogares sin sanitario, sin acueducto o que teniendo acueducto utilizaran agua de río, nacimientos, quebradas, carrotanques, lluvia, aguateros. En zona rural: sin sanitario, ni acueducto y que tomaran agua de lluvia, aguateros, embotelladas, río, quebrada, manantial o nacimiento
	Alcantarillado	Categórica	
	Fuente de agua para preparar los alimentos	Categórica	
Dependencia económica	Relación de parentesco con el jefe de hogar	Categórica	Más de tres personas por miembro ocupado y el jefe del hogar tenga como máximo dos años de educación primaria aprobados
	Número de personas en el hogar	Continua	
	Ocupación en la semana previa al cuestionario	Categórica	
	Nivel educativo más alto alcanzado y último año o grado aprobado en ese nivel	Categórica	
Inasistencia escolar	Edad	Continua	Niños en edades entre los seis y los doce años familiares del jefe del hogar que no asistían a un centro educacional
	Asistencia escolar	Categórica	
	Parentesco con el jefe de hogar	Categórica	

Fuente: construcción a partir de Castro Alfaro *et al.*^3^.

El procesamiento y análisis fue soportado por Microsoft Office Excel para Mac, versión 16.54, y el *software* estadístico IBM SPSS Statistics versión 27.

En la dimensión de inasistencia escolar las edades utilizadas en el cálculo fueron de 5 a 14 años, dada la conformación quinquenal de los datos del CNPV 2018. En la dimensión alta dependencia económica, en la variable nivel educativo más alto alcanzado o grado aprobado en ese nivel, se utilizaron datos de preescolar y ningún estudio. Los hogares se clasificaron en tres categorías: hogar con todas las necesidades satisfechas; hogar con una necesidad no satisfecha, es decir, un hogar en situación de pobreza; y hogar con dos o más necesidades insatisfechas, es decir, un hogar en situación de miseria 15.

Al realizar la prueba de Kolmogorov-Smirnov, los datos no tenían una distribución normal y había sobre-dispersión, por lo cual se realizó un análisis de regresión binomial negativa para estimar la asociación de la pobreza y el sobrepeso/obesidad infantil en cada municipio de la Región Pacífica y Bogotá D. C., asumiendo como variable dependiente la prevalencia de obesidad en el 2018 y como variables independientes la pobreza, la miseria y las necesidades básicas satisfechas. Los resultados de las regresiones binomiales se expresaron como Odds Ratio (OR) con intervalos de confianza del 95 % (IC 95 %), considerando un nivel de significancia de 0,05 para todos los análisis.

El estudio no fue considerado por un comité de ética, debido a que las fuentes de información consolidadas y analizadas son generadas por instituciones públicas y de acceso libre, lo cual no tiene impactos bioéticos o ambientales.

## RESULTADOS

En Colombia, entre el 2015 y el 2019, en los servicios de consulta externa y urgencias se atendió por obesidad/ sobrepeso a 340 944 NNA entre los 5 y los 19 años. El promedio de atención fue de dos consultas por año. Durante este periodo, el costo de atención médica a personas entre 0 y 100 años en los servicios de urgencias y consulta externa fue de USD $90 608 422, de los cuales USD $8 521 246 (9,4 %) se destinaron a la atención de sobrepeso/obesidad en el grupo de 5 a 19 años, es decir, que cada atención a NNA tuvo un valor de USD $25 dólares.

Para el 2018, en la ciudad de Bogotá D. C., el 6,8 % y el 0,5 % de los NNA entre 5 y 19 años estaban en situación de pobreza y miseria, respectivamente, y la prevalencia de sobrepeso/obesidad en esta población fue de 1 127 casos por 100 000 habitantes. En la Región Pacífica, para el 2018, la prevalencia más alta de sobrepeso/obesidad en NNA se encontró en el Valle del Cauca, donde los porcentajes de NNA en hogares en situación de pobreza y miseria fueron menores que en los otros departamentos de la región. En el Chocó, donde el porcentaje de NNA en hogares en situación de pobreza o miseria fue el más alto de la región, se encontró que la prevalencia de obesidad era inferior a la encontrada en el Valle del Cauca o Bogotá D. C. para el mismo periodo, como se indica en la Tabla 2.

**Tabla:** 2. Prevalencia de obesidad y porcentaje de NBI, según área de residencia, Colombia, 2018

Área de residencia	Prevalencia obesidad 2018	Necesidades básicas satisfechas	Pobreza	Miseria
Bogotá	1127	92,7	6,8	0,5
Valle del Cauca	741	90,3	9,0	0,7
Cauca	398	78,3	17,7	4,0
Chocó	385	36,8	36,5	26,8
Nariño	133	73,6	21,1	5,3

Fuente: construcción a partir de datos del Censo Nacional de Población y Vivienda (CNPV) 14 e información registrada en la Bodega de datos Sispro, RIPS 13.

### Efecto de la pobreza en la prevalencia de sobrepeso/ obesidad

En Bogotá D. C., el modelo de regresión binomial negativa no arrojó datos con significancia estadística entre la prevalencia de sobrepeso/obesidad y el NBI (OR 1 (IC 95 % 0,97-1,0); P=1,0). En la Región Pacífica, los datos fueron estadísticamente significativos (P < 0,05) al estimar el efecto del NBI en el sobrepeso/obesidad, según el cual, en los departamentos de Cauca, Valle del Cauca, Nariño y Chocó, aquellos NNA que vivían en situación de pobreza en el 2018 tuvieron un riesgo menor de tener sobrepeso/ obesidad, en comparación con aquellos que vivían en hogares sin necesidades básicas descubiertas. Los NNA que viven e hogares en condición de pobreza residentes en Nariño (OR=1,6) y Chocó (OR = 1,5) tuvieron un riesgo superior de sobrepeso/obesidad que aquellos que residían en los departamentos de Cauca (OR = 1,3) y Valle del Cauca (OR=1,3), donde el riesgo fue similar, como se indica en la Tabla 3.

**Tabla 3:** Análisis de la correlación entre los componentes de NBI y la obesidad infantil, Región Pacífica y Bogotá, 2018

Ubicación	Variable		B	OR	IC 95 %		P-valor
					Inferior	Superior	
Bogotá	Necesidades básicas	Satisfechas	-2,8E-16	1	0,9	1,0	1
		Pobreza	2,7E-16	1	0,9	1,0	1
		Miseria	0a	1	.	.	.
Valle del Cauca	Necesidades básicas	Satisfechas	0,3	1,3	1,3	1,4	0,0
		Pobreza	0,2	1,2	1,2	1,3	0,0
		Miseria	0a	1	.	.	0,0
Cauca	Necesidades básicas	Satisfechas	0,5	1,6	1,5	1,6	0,0
		Pobreza	0,2	1,3	1,2	1,3	0,0
		Miseria	0a	1	.	.	.
Nariño	Necesidades básicas	Satisfechas	0,8	2,3	2,2	2,3	0,0
		Pobreza	0,5	1,6	1,6	1,7	0,0
		Miseria	0a	1	.	.	.
Chocó	Necesidades básicas	Satisfechas	0,4	1,6	1,5	1,6	0,0
		Pobreza	0,4	1,5	1,5	1,5	0,0
		Miseria	0a	1	.	.	.

Fuente: construcción a partir de datos del Censo Nacional de Población y Vivienda (CNPV) 14 e información registrada en la Bodega de datos SISPRO, Sistema de Gestión de Datos - SGD - Cubo RIPS. Fecha de consulta: 9 de noviembre de 2021. 13.

## DISCUSIÓN

Este estudio muestra que la pobreza es un factor de riesgo para el sobrepeso/obesidad, sin embargo, los NNA que viven en situación de pobreza tienen una menor probabilidad de ser diagnosticados con sobrepeso/obesidad que aquellos que viven en hogares en capacidad de suplir todas las necesidades básicas. El haber trabajado con el universo de la población permitió deducir que los datos encontrados realmente son representativos de las poblaciones estudiadas.

En América Latina y Colombia, las cifras de sobrepeso/ obesidad van en aumento. Según la ENSIN-2010, se estima que uno de cada dos colombianos tiene exceso de peso 12, lo cual es acorde a lo encontrado en esta investigación, al observar un aumento en la prevalencia de sobrepeso/obesidad del 2015 al 2019.

En los departamentos con hogares con mayor porcentaje de necesidades básicas cubiertas, la prevalencia de sobrepeso/obesidad fue mayor que en aquellos con necesidades básicas insatisfechas; ejemplo de ello es el Valle del Cauca, el cual reportó una prevalencia de sobrepeso/obesidad mayor a los otros departamentos. Sin embargo, dada la naturaleza de la información recolectada, no fue posible determinar si las barreras de acceso que limitan la atención medica constituyeron la razón por la cual los otros departamentos reportaron una prevalencia menor de sobrepeso/obesidad.

De la población total de estudio, con edades comprendidas entre los 5 y los 19 años en el 2018, el 48 % residía en Bogotá D. C. (1 447 512) y el 52 % en la Región Pacífica, en la cual el número de NNA en condiciones de pobreza y miseria superó al de la ciudad capital. La distribución desigual de las condiciones de vida puede afectar a su vez la distribución del sobrepeso/obesidad u otras patologías, dado que el contexto social tiene un papel importante en el estado de salud de las personas (14), como fue demostrado por Álvarez, quien reportó una relación positiva entre el bajo nivel socioeconómico y la obesidad en adultos 16.

En el presente estudio los resultados indicaron que, en el 2018, en los departamentos del Valle del Cauca (P<0,05), Chocó (P>0,05) y Nariño (P>0,05), el riesgo de obesidad aumentó en los hogares que no estaban en condición de pobreza, lo cual difiere con lo reportado por Rivera *et al.*^10^, quienes a partir de la Encuesta Nacional de Salud en México reportaron que los niveles de obesidad/sobrepeso disminuían a medida que las condiciones socioeconómicas mejoraban, coincidiendo con lo descrito por Morales-Ruán *et al.*^17^.

En Bogotá D. C. no se encontró asociación entre el NBI y el sobrepeso/obesidad, sin ser estadísticamente significativo. Esta falta de correlación puede deberse a la protección de los cuidadores, como fue mencionado por Dinour *et al.*^16^, quienes encontraron que la ausencia de correlación entre la inseguridad alimentaria y el sobrepeso en los niños podía ser fruto de la reasignación de recursos por parte de los cuidadores, como manera de protegerlos frente a situaciones de inseguridad alimentaria. Morales-Ruán *et al.*^17^ también reportaron la relación inversa entre sobrepeso/obesidad y estado socioeconómico en las mujeres adultas.

Dinour *et al.*^18^ concluyeron que los resultados inconsistentes entre la asociación de sobrepeso/obesidad y la inseguridad alimentaria en NNA se veían influidos por otras variables como la edad, la raza, la etnia, los ingresos familiares, el sexo y la definición de inseguridad alimentaria, las cuales se deben tener en cuenta en un próximo estudio durante la recolección y el análisis de los datos 16. Esto, también fue concluido por Shariff y Khor 19 en un estudio realizado en hogares rurales de Malasia.

### Limitaciones

Al tratarse de un estudio ecológico, la principal limitación consiste en que no se pueden identificar asociaciones a nivel individual, dado que al hacerlo se podría incurrir en una falacia ecológica 20. Otra limitación se atribuye al uso de fuentes secundarias, sin poderse obtener datos adicionales, lo que habría podido enriquecer el análisis. Además, no se estimó la influencia de la clase social o la etnia en el diagnóstico de sobrepeso/obesidad, y al no contar con el índice de masa corporal, no se distinguió entre sobrepeso y obesidad, por lo cual durante esta investigación estos dos diagnósticos se trataron indistintamente. Adicionalmente, no se establecieron correlaciones con comorbilidades existentes en NNA atendidos por sobrepeso/obesidad en este periodo.

El promedio de atenciones fue de dos consultas anuales, según la relación entre número de atenciones y número personas atendidas. Sin embargo, no se determinó si uno de estos NNA recibió más o menos atenciones en el año, ni se identificó si en un hogar donde hay más de un menor, todos fueron diagnosticados con sobrepeso/obesidad.

El NBI no permite identificar el nivel de intensidad de pobreza, ni es útil para identificar situaciones de pobreza reciente, es decir, hogares que tienen todas sus necesidades satisfechas, pero no cuentan con un ingreso suficiente para adquirir bienes o servicios básicos como los alimentos y llevar una dieta adecuada. Esto cobra importancia en el actual escenario de pandemia por COVID-19, en el cual disminuyeron los ingresos de los hogares en el mundo.

Para futuros estudios se sugiere la inclusión de variables como la influencia cultural en la composición y variedad de la dieta, la etnia, los ingresos y los gastos del hogar en alimentación, medidas antropométricas (talla, peso), si es beneficiario o no de algún programa de ayuda alimentaria, actividad física en el tiempo libre, para determinar su influencia en relación con el sobrepeso/ obesidad. Además, se sugiere la realización de estudios cualitativos que aborden de qué manera se suple la alimentación en situaciones de privación económica y si hay una reasignación de recursos para proteger a algunos miembros del grupo familiar.

La obesidad como fenómeno multicausal, por su magnitud y consecuencias, ha trascendido lo individual para convertirse en un problema social, y constituye un desafío para la salud pública a escala mundial.

En este estudio, la elección de datos agregados de los departamentos de la Región Pacífica y Bogotá D. C. como unidad de observación permitió evaluar los niveles de exposición a las variables tomadas como independientes en el diagnóstico de sobrepeso/obesidad.

La diferencia en el porcentaje de hogares de NNA que viven en situación de pobreza y miseria entre los departamentos de la Región Pacífica (Chocó: 63,3; Cauca 21,7; Nariño: 7,2 y Valle del Cauca: 0,7 %) y la ciudad capital (7,3 %) refleja la desigualdad que existe entre las diferentes zonas del país, lo cual requiere un abordaje e intervención diferente del problema del sobrepeso/obesidad infantil a nivel de políticas públicas en estas poblaciones.

Se encontró una relación positiva entre la pobreza y el sobrepeso/obesidad en los departamentos de la Región Pacífica, sin poderse demostrar que el vivir en condiciones de pobreza aumentara el riesgo de tener un desenlace de sobrepeso/obesidad mayor a aquellos que no lo estuvieran. Por lo anterior, se considera que, para esclarecer la relación entre variables socioeconómicas, como la pobreza y el sobrepeso/obesidad, es necesario tener en cuenta variables adicionales como la clase social, la etnia y todas aquellas que influyan en el comportamiento individual, más allá de los ingresos económicos familiares o personales *
